# Breast Cancer Metastasis to the Optic Nerve: A Case Report

**DOI:** 10.7759/cureus.91984

**Published:** 2025-09-10

**Authors:** Othmane Zouiten, Fatima Zahra Abbassi, Leila Afani, Mohamed El Fadli, Rhizlane Belbaraka

**Affiliations:** 1 Medical Oncology, Cadi Ayyad University Faculty of Medicine and Pharmacy of Marrakech, Marrakech, MAR

**Keywords:** breast cancer, invasive lobular breast carcinoma, ophthalmologic presentation, optic nerve metastasis, vision loss

## Abstract

Optic nerve metastases from breast cancer are an extremely rare event, with very limited cases reported in the literature. In this report, we present the case of a 64-year-old woman with a history of hormone-receptor-positive, human epidermal growth factor receptor 2 (HER2)-negative invasive lobular carcinoma of the breast, who developed bilateral progressive vision loss almost 10 years after her initial diagnosis. Despite previous treatment using surgery, chemotherapy, radiotherapy, and hormone therapy, the progression of the disease included brain, lung, and bone metastases. The patient suffered from blurred vision that rapidly worsened, leading to complete blindness. Bilateral optic atrophy was noted on ophthalmological evaluation, and MRI findings included nodular thickening and enhancement of the pre-chiasmatic optic nerves, which were consistent with metastatic involvement. Due to her poor general condition, she was put on weekly paclitaxel systemic chemotherapy, with no indication for surgery. This is a unique case, as it highlights optic nerve metastasis as a late manifestation of lobular breast cancer and underlines the significance of considering metastatic disease in patients with newly presenting visual symptoms and a history of breast cancer. The main lesson to be learned from this report is that early detection and multidisciplinary evaluation are crucial to optimize the patient's care, despite the fact that therapeutic options may be limited at an advanced stage of the disease.

## Introduction

Breast cancer is the most commonly diagnosed malignancy and the leading cause of cancer-related death among women worldwide. Its natural history is markedly variable, characterized by a significant risk of metastasis to distant organs, which remains the primary determinant of prognosis and survival. The most common sites of metastasis include the bone, liver, lungs, and brain, reflecting the disease's heterogeneous and aggressive potential [1].

Within the spectrum of cerebral involvement, metastases to the orbit and ocular region are already a rare occurrence, accounting for a small fraction of all secondary tumors. Among these, optic nerve metastasis represents an exceedingly uncommon and clinically profound phenomenon. With only a select number of cases documented in the published literature, optic nerve metastasis is often a devastating diagnostic finding, typically signaling advanced, widely disseminated disease [2].

This case report contributes to the growing yet limited literature on the rarity of optic nerve metastases in breast cancer patients. It underscores the critical importance of maintaining a high index of clinical suspicion for atypical neurological and ophthalmological presentations in oncology patients. Furthermore, it highlights the diagnostic challenges inherent in detecting such lesions and emphatically underlines the necessity for rapid, multidisciplinary intervention. Early detection and timely treatment are paramount not only in attempting to preserve visual function - a crucial aspect of quality of life - but also in potentially altering the disease course and improving overall prognosis for these patients.

## Case presentation

A 64-year-old woman reported gradually deteriorating blurred vision in both eyes. In 2014, she had been initially diagnosed with invasive lobular carcinoma of the right breast after experiencing retraction of the nipple and bloody discharge. She has no personal or family history of cancer and does not have any children. The patient is post-menopausal and used to be a full-time homemaker.

At the initial diagnosis, a thoraco-abdomino-pelvic computed tomography (CT) scan showed no signs of metastasis. She was operated on with a mastectomy of the right breast with axillary curage. Pathological findings revealed a Scarff-Bloom-Richardson (SBR) grade II infiltrating lobular carcinoma, with involvement of the nipple-areolar plate, and were associated with edema and orange peel skin, the existence of lymphovascular emboli, and perineural infiltration. Tumour staging was pT4b N0 M0, corresponding to a clinical stage IIIB, indicative of a locally advanced tumour with neither lymph node invasion nor distant metastases. Immunohistochemistry revealed that the tumor was positive for both estrogen receptor (ER) and progesterone receptor (PR) and negative for human epidermal growth factor receptor 2 (HER2-neu).

After surgery, she underwent adjuvant chemotherapy (three courses of epirubicin at a dose of 100 mg/m^2^ plus cyclophosphamide at a dose of 600 mg/m^2^, followed by three courses of docetaxel at a dose of 75 mg/m^2^), followed by 50 Gy of external radiotherapy and five straight years of letrozole-based hormonal therapy.

In 2022, she subsequently developed a cerebral metastasis, which was operatively excised and treated with 10 sessions of radiotherapy with a total of 30 Gy. The metastatic lesion had an identical molecular profile to the initial breast tumor.

**Figure 1:**
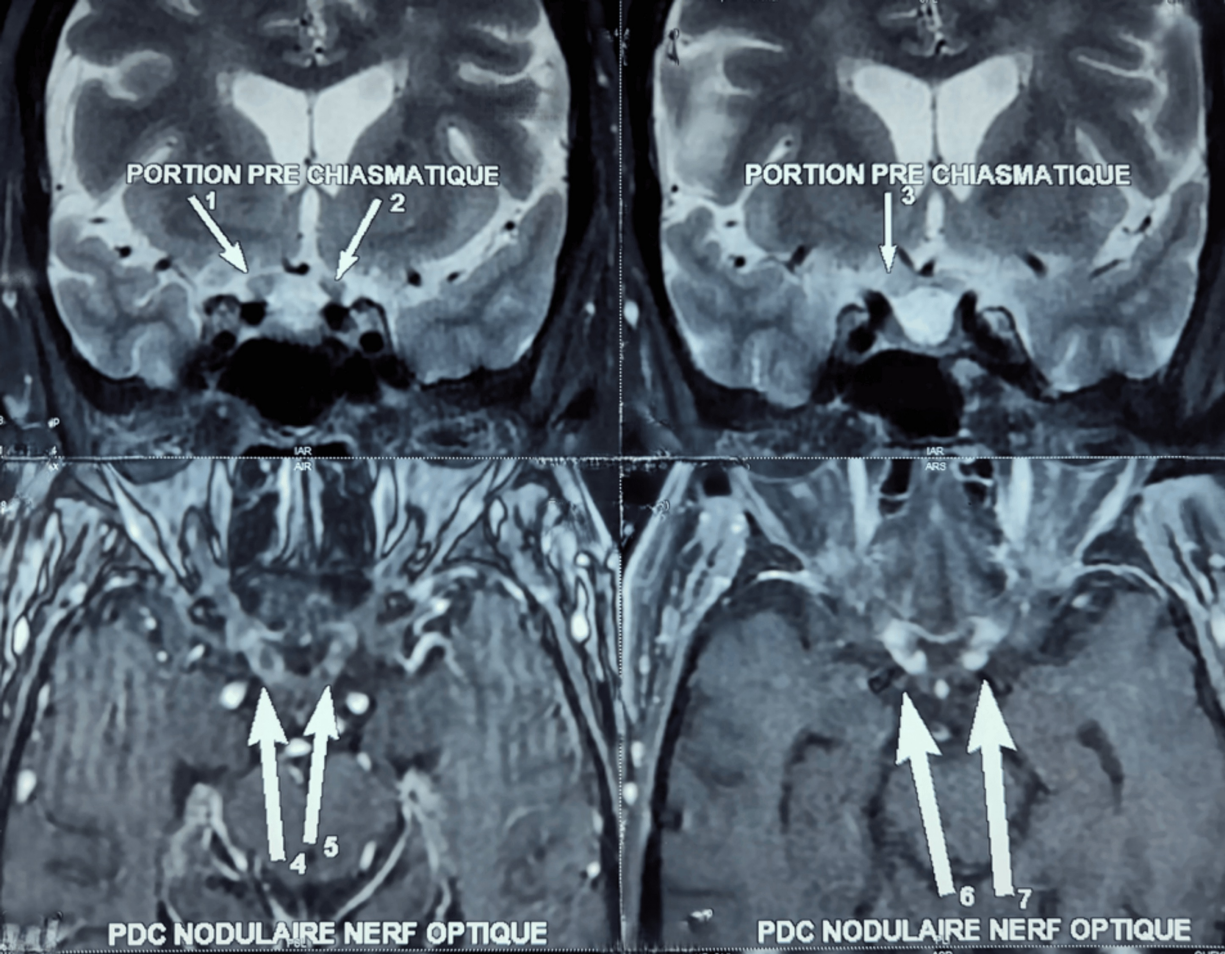
MRI images showing optic nerve thickening

In December 2024, she progressed to lung and bone metastases, and her visual acuity quickly decreased, resulting in total bilateral blindness. Magnetic resonance imaging (MRI) (Figure 1) revealed thickening and nodular enhancement of the optic nerves in their intracranial prechiasmatic segments, consistent with metastatic disease. MRI showed no signs of carcinomatous meningitis, in particular, no leptomeningeal or dural enhancement and no communicating hydrocephalus. The diagnosis of optic nerve metastases was confirmed based on the imaging findings and the patient's clinical history.

**Figure 2:**
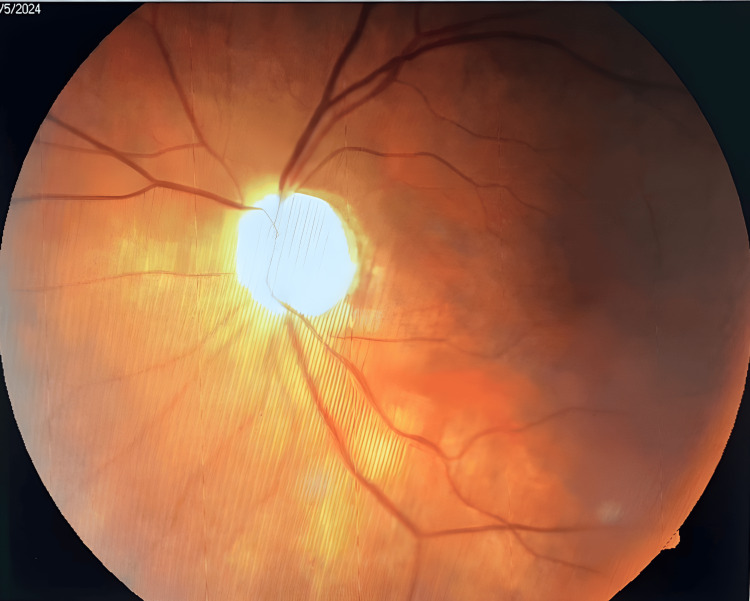
Right eye fundus image

**Figure 3:**
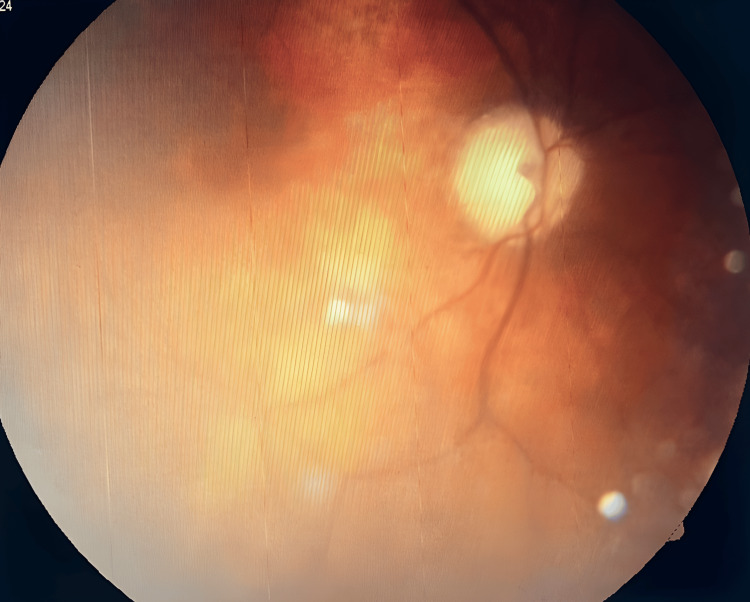
Left eye fundus image

Ophthalmological investigation demonstrates a completely absent perception of light in either eye, whether corrected or uncorrected, reflecting total bilateral blindness. The pupillary reaction to light (PRL) is present despite a dilated pupil, which suggests a persistent pupillary anomaly. Extra-ocular muscular movements are complete, without noticeable proptosis. Ocular tension (OT), measured by the contact billan, is in the normal range for both eyes, which rules out ocular hypertonia or acute glaucoma. The ocular adnexae (eyelids, conjunctiva, lacrimal apparatus) are all normal. Slit-lamp exam reveals a clear cornea and anterior chamber (AC) of correct depth in both eyes, a normal iris with no signs of neovascularization or atrophy, and a bilateral cortical cataract in the lens. Whilst this cataract is likely to be a contributing factor in the visual deficit, it does not seem to be the main cause of the blindness encountered, considering the absence of perception of light. Fundus examination (Figures 2, 3) reveals bilateral atrophy of the optic nerve, indicating considerable damage to the optic nerve. The fundus also shows evidence of retinal choroidal arteriosclerosis (RCA); these vascular changes also include the macula, seemingly embedded in these arterial changes, and a retina that has remained flat.

Given the disease's aggressive nature, poor immediate prognosis, and ineligibility for local treatment, we diagnosed an imminent visceral crisis. Consequently, the patient was put on first-line chemotherapy with a weekly course of Paclitaxel, a decision driven by the advanced stage of her disease, which made hormone therapy combined with a CDK4/6 inhibitor an unsuitable initial option.

The patient tolerated the eight-week course of paclitaxel well; however, her death ensued following a massive pulmonary embolism.

## Discussion

Ocular metastases from carcinomas are rare, with an incidence ranging from 1% to 12.6%. Among these, breast cancer is the most common primary source. Involvement of the optic nerve itself is exceptionally uncommon, often misdiagnosed, and typically appears alongside choroidal or other systemic metastases. Isolated optic nerve lesions are remarkably rare, emphasizing the need for clinicians to consider metastatic disease when patients with a cancer history present with optic nerve anomalies, as these symptoms are easily mistaken for more common ophthalmological conditions [2].

The pathophysiology of optic nerve metastasis is believed to be hematogenous, with tumor cells spreading through the blood supply. The optic disc is often affected by extension from a choroidal metastasis due to the shared vascular network, although isolated nerve involvement can occur. Presenting symptoms are non-specific, including blurred vision, visual field defects, eye pain, and changes in intraocular pressure, which frequently lead to diagnostic delays and confusion with benign conditions [3-4].

These metastases can manifest at any stage of breast cancer, from the initial diagnosis to many years later. The interval between the primary cancer diagnosis and ocular metastasis can vary widely, from one month to over 25 years, with recent studies indicating a median of 4.5 to 6.5 years [5]. Notably, the invasive lobular carcinoma subtype appears to have a distinct metastatic pattern and may be more prone to spreading to rare sites like the optic nerve compared to the more common invasive ductal carcinoma [6].

Diagnosis requires a thorough ophthalmological evaluation, including visual acuity tests, slit-lamp examination, and imaging such as ultrasound, CT, or MRI [7]. A multidisciplinary approach involving oncologists, radiologists, and ophthalmologists is crucial for both diagnosis and management. This collaboration is especially critical given that the location of these metastases often makes a tissue biopsy impossible or prohibitively risky. Therefore, the diagnosis frequently relies on a combination of clinical presentation, characteristic imaging findings on MRI or CT, and the context of a known systemic malignancy, underscoring the need for expert radiological and ophthalmological interpretation [8].

Treatment options, including radiotherapy or surgery, aim to preserve vision and alleviate symptoms but depend on the tumor's location, the extent of systemic disease, and the patient's overall prognosis [9]. The rarity of this condition limits evidence-based protocols, making treatment decisions challenging and often reliant on individualized case management developed through multidisciplinary tumor board consensus [10].

## Conclusions

Optic nerve metastases from breast cancer are quite rare and are often a sign of advanced disease with a poor prognosis. Its symptoms are nonspecific and make diagnosis extremely challenging, necessitating a multidisciplinary approach for optimal management. While early recognition is crucial, treatment options remain limited, particularly in advanced cases. We believe this case contributes to raising awareness of this infrequent metastatic disease among healthcare professionals. Increased acknowledgement and reporting of such cases could help to improve the accuracy of diagnosis and guide future management strategies for similarly affected patients.
